# 24-h Urine Collection: a Relevant Tool in CKD Nutrition Evaluation

**DOI:** 10.3390/nu12092615

**Published:** 2020-08-27

**Authors:** Moustafa Abdel-Nabey, Camille Saint-Jacques, Jean-Jacques Boffa, Vincent Frochot, Marine Livrozet, Michel Daudon, Martin Flamant, Emmanuel Letavernier, Jean-Philippe Haymann, on behalf of the NephroTest Study Group

**Affiliations:** 1 Service d’Explorations Fonctionnelles Multidisciplinaires, AP-HP, Hôpital Tenon, Sorbonne Université, 75020 Paris, France; camille.saint-jacques@aphp.fr (C.S.-J.); vincent.frochot@aphp.fr (V.F.); marine.livrozet@aphp.fr (M.L.); michel.daudon@aphp.fr (M.D.); emmanuel.letavernier@aphp.fr (E.L.); 2 INSERM, UMR_S 1155, AP-HP, Hôpital Tenon, Sorbonne Université, 75020 Paris, France; jean-jacques.boffa@aphp.fr; 3 Service de Néphrologie, AP-HP, Hôpital Tenon, Sorbonne Université, 75020 Paris, France; 4 Department of Physiology, University Paris Descartes-Paris 5, AP-HP, Hôpital Bichat, 75018 Paris, France; martin.flamant@aphp.fr

**Keywords:** 24 h-urine collection, chronic kidney disease, nutrition, salt, diet protein intake

## Abstract

Dietary management is a cornerstone of Chronic Kidney Disease (CKD) monitoring, and dietary surveys often difficult to perform. We studied in a CKD patient cohort with two years follow-up, whether validated 24-h urine ionogram would be a relevant tool for diet evaluation and compliance. We included 404 non-dialysis CKD patients, with three evaluations, including repeated measurements of fractional renal creatinine clearance and 24-h urine collection. Completeness of the 24-h urine collection, assessed by daily urine creatinine excretion extrapolated from fractional creatinine clearance, was 64.6%, 75.5%, and 78.2% at the first, second, and third visits, respectively. One hundred sixty-eight patients (41.6%) had three complete collections, with a measured glomerular filtration of 42.3 mL/min/1.73 m^2^ at baseline and prevalence of anemia and secondary hyperparathyroidism of 13.9% and 26.2%, respectively, increasing during follow-up to 15% and 31.5% (*p* < 0.001 and *p* < 0.001). The urine analysis showed at baseline a urine volume of above 2 L/day, and estimated sodium and protein intake within targets in 51.6% and 40.3% of cases, which improved during follow-up only for protein (to 45.9%, *p* < 0.0001). Our data suggest that a 24-h urine ionogram is an interesting, reliable tool in CKD patients for dietary monitoring to achieve target recommendation noteworthy salt and protein intake.

## 1. Introduction

Chronic kidney disease (CKD) is a public health problem worldwide with prevalence between 8% and 16% [1] responsible for significant cardiovascular morbidity and mortality [2,3]. The glomerular filtration rate (GFR) decline varies with aging and kidney diseases [4], but also nonspecific CKD risk factors such as uncontrolled hypertension, uncontrolled diabetes, and proteinuria [5,6].

Dietary management is a cornerstone of CKD monitoring with two main focuses: (1) To control metabolic complications related to CKD such as metabolic acidosis, hyperkalemia, hyperphosphatemia, and secondary hyperparathyroidism [7]; (2) To slow GFR decline by reducing protein load diet and sodium intake. Indeed, the Mediterranean diet [8] is recommended in CKD patients, according to KDIGO (Kidney Disease Improving Global Outcomes) 2012 [7] altogether with a low-sodium and low-protein diet. A low-sodium diet is indeed the main target in order to better control blood pressure, which is a relevant risk factor for cardiovascular events [9], whereas a high sodium intake is indeed considered deleterious both on blood pressure control and CKD progression due to remnant nephrons hyperfiltration [10]. Moreover, low protein diet prescribed to non-end-stage renal disease patients could–in addition to preserving renal function–be an important strategy to prevent disturbed gut microbiota reported in CKD patients and thus decrease levels of uremic toxins (such as indoxyl sulfate) and cardiovascular risk factors [11,12,13]. A controlled diet is also aimed at decreasing potassium intake to prevent hyperkalemia and increase calcium and vitamin D intake in order to delay secondary hyperparathyroidism [14]. Indeed, in CKD stage III–V, a lower calcium gut absorption (related to vitamin D hydroxylation impairment) may be detrimental to bone mass [15]. However, diet inquiries are expensive and time-consuming, and monitoring remains uncertain due to the lack of specific tools. Though available surveys, such as the Dietary Sodium Restriction Questionnaire [16,17], are prone to favor the understanding of patients on low sodium diet, their accuracy in evaluating sodium intake may be questionable in some cases. Therefore, the use of a relevant and easy-to-perform tool to quantify salt and diet protein intake “at the bedside” and at lower cost is most welcome. 

Performing 24-h urine collection could alternatively or in addition to follow-up by dieticians, be a simple and inexpensive tool to assess objectively (if the patient is in a steady-state) daily sodium and protein intakes, and thus compliance to dietary targets [18]. However, this potential tool remains underused due to the current view that 24-h urine collection is difficult to obtain—including incomplete 24-h collection due to misunderstanding (if two nights are included or if some voiding’s are missing), and thus not reliable in many cases [19,20]. In previous work from the Nephrotest cohort study group, we provided expected 24-h urine creatinine normal range values expressed as µmol/kg body weight, according to gender, age, and CKD stages [21]. Though 24-h urine creatinine values provide an estimate of muscle mass, it may also validate complete 24-h voiding collections. 

The objective of the present work is to study the reliability of 24-h urine collection and diet compliance obtained by a validated 24-h urine ionogram in a well-characterized non-end-stage CKD cohort over a two year follow-up period.

## 2. Patients and Methods

### 2.1. Ethics Statement 

All patients signed written informed consents before inclusion in the cohort. The NephroTest study design was approved by an ethics committee (Direction générale pour la recherche et l’innovation. Comité consultatif sur le traitement de l’information en matière de recherche dans le domaine de la santé (CCTIRS). Ref: DGRI CCTIRS MG/CP09.503, 9 July 2009).

### 2.2. Study Concept

**Figure 1:**
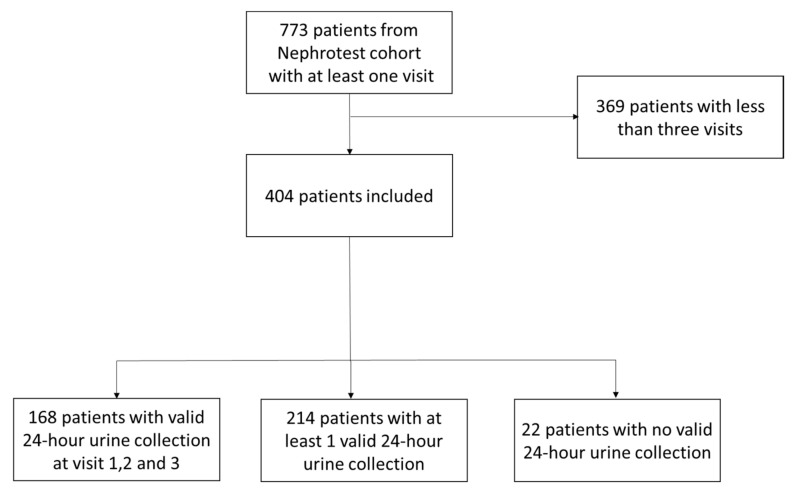
Flow chart of the study sample.

The NephroTest study is a prospective hospital-based cohort that began in 2000; by the end of 2010, it had enrolled 1827 adult patients with all stages of CKD and all nephropathy types referred by nephrologists to any of the three departments of physiology for annual extensive workups. Eligible patients were 18 years of age at inclusion and had neither started dialysis nor received a kidney transplant. Pregnant women were excluded. This retrospective study included 773 patients followed in hospital Tenon (Paris) between 2005 and 2011 from the Nephrotest prospective cohort. In order to have a longitudinal follow-up, we included 404 patients with at least three out-patient hospitalizations one year apart (i.e., at least three visits) (Figure 1). The patients in this cohort benefited from out-patient hospitalizations lasting six to seven hours. During this course, they underwent a clinical examination, a biological workup (blood and urine, including 24-h urine collection), and a measured glomerular filtration rate (mGFR) using ^51^Cr EDTA (chromium-51 labeled ethylenediamine tetraacetic acid) renal and plasmatic clearance associated with creatinine clearance (fractional creatinine clearance) during six periods of 30 min. Mean values of mGFR and fractional creatinine clearance performed on six sequential spot urine samples were recorded.

At the time of out-patient hospitalization, subjects also provided 24-h urine collection, allowing 24-h urine creatinine measurement (Jaffe method) and 24-h creatinine clearance. Since the 24-h urine collection may be inaccurate, we validated urine collection through creatininuria, and therefore, estimated that a maximum delta of 30% between the measured 24-h urine creatinine and 24-h urine creatinine excretion extrapolated from fractional creatinine clearance was acceptable, on the assumption that creatinine excretion is stable over the 24-h period. In other words, we considered a 30% threshold between measured 24-h creatinine clearance and the mean fractional creatinine clearance. For outliers, the urinary collection was considered too important to be considered reliable. 

Urinary urea, expressed as mmol/24-h was also expressed as protein intake/kg body weight/day (24-h urea × 0.2/kg body weight). In order to compare a protein diet for a given patient during follow-up, despite weight gain or loss, the results were normalized for a body mass index of 22 kg/m^2^. Twenty-four-hour urine sodium reflecting sodium intake at steady-state was also expressed as sodium chloride intake (24-h natriuresis divided by 17). Twenty-four-hour urine osmolarity was measured by an osmometer 3320 (Advanced Instruments Inc., Norwood, MA, USA). Anemia was defined by a Hb < 13 g/dL and < 12 g/dL for males and females, respectively (7). Serum parathyroid hormone (PTH, normal values: 12–65 pg/mL) was measured by radioimmunoassay (ELSA PTH CISBIO Kit, 30200 Codolet/France). Missing values accounted for less than 5% of most indicators.

### 2.3. Statistical Analyses 

Variables were expressed as percentages, means +/− standard deviation (SD), or medians +/− interquartile range (IQR), as appropriate. Comparison between the two groups was performed with three validated 24-h urine collections versus no validated urine collection at all visits using a non-parametric Mann–Whitney test or the χ^2^ test when appropriate. Comparison of clinical, biological, and dietary parameters between the three visits in patients with all validated 24-h urine collections was performed using a non-parametric Friedman test. Statistical analyses were performed with the Statview software, version 5.01 (SAS institute Inc., Cary, NC, USA). A *p* value < 0.05 was considered statistically significant.

## 3. Results

**Figure 2:**
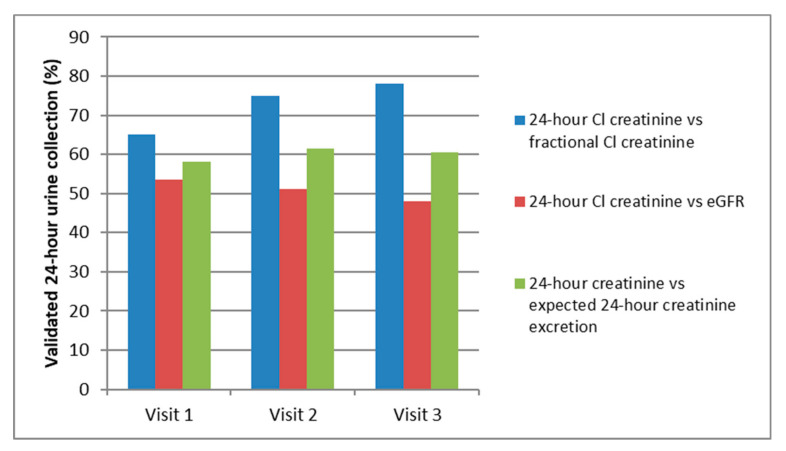
Prevalence of validated 24-h urine collection for each visit, according to fractional creatinine clearance, eGFR (estimated glomerular filtration rate), or expected 24-h creatinine excretion (18).

Among 773 patients from the Nephrotest cohort included in our center, 404 patients with at least three evaluations with a median interval between visits of 1.12 year (1.00–1.69) were included (Figure 1). At the first visit, 261 patients (65%) had a valid 24-h urine collection defined as a difference below 30% between the mean fractional creatinine clearance performed on six sequential spot urine samples and 24-h urine sample. As shown in Figure 2, 305 patients (75.5%) had a valid 24-h collection at the second visit and 316 at the third visit (78.2%). Overall, 168 patients had complete urine collections for the three visits whereas only 22 had no reliable urine collection at all. Interestingly, at the first visit, 58 patients out of 404 (14.4%) had more than 24-h urine collection samples and 85 (21%) had incomplete collections. At the third visit, patient education improved the results (with 16 (4%) collections in excess and 72 (17.8%) incomplete collections). Prevalence of valid 24-h urine collection was 50.7% and 67.1% at visit 1 and 3, respectively, when using a more stringent threshold of 15%. Moreover, as shown in Figure 1, prevalence of valid 24-h urine collection was lower, around 50% (with no increase during follow-up) when considering a difference below 30% between the estimated GFR value by MDRD (Modification of Diet in Renal Disease) equation and creatinine clearance performed on a 24-h urine sample. Of notice, a prevalence of 60% of valid 24-h urine collection was found using an expected 24 h-urine creatinine excretion median value, according to the GFR level, gender age, and ethnicity (18).

When comparing patients with complete urine collections for all three visits (*n* = 168) with patients with no reliable urine collection at all visits (*n* = 22), the two groups look similar for age and gender (60.8 versus 61.8 years, with 48.8% and 36.4% of men, respectively) and cardiovascular risk factors with a prevalence of 28.1% versus 14.3% of diabetes (*p* = 0.43), and 100% versus 90.6% of hypertension (*p* = 0.4). The body mass index was higher in patients with no valid 24-h urine collection compared to patients with three valid urine collections (31.6 versus 26.5 kg/m^2^, *p* = 0.003). As shown in Table 1, the mGFR as well as mean fractional creatinine clearance or serum creatinine, were similar. Of interest, the lack of 24-h urine collection compliance was not associated with more CKD metabolic complications such as hyperkalemia, anemia, hypocalcemia, or hyperparathyroidism (Table 1). As expected, daily diuresis and urine creatinine were significantly different between the two groups with lower figures in the latter group suggesting incomplete urine collection and a potential lack of compliance. A lower amount of urine albumin or protein is in accordance with these findings. However, of notice, the albumin/creatinine ratio (ACR) and protein/creatinine ratio were similar between the two groups. 

As shown in Table 2, in patients with complete urine collections for all three visits at baseline (*n* = 168), the median mGFR was 42.3 mL/min/1.73 m^2^, and anemia and secondary hyperparathyroidism were present in 46.1% and 26.2% of cases, respectively. As shown, the prevalence of secondary hyperparathyroidism increased up to 31.5% during follow-up. Despite an mGFR average decrease of 2.6 mL/min/year, an increased ACR and a trend for an increased weight between each visit, prevalence of blood pressure below target values of 130/80 mmHg didn’t increase. Of notice, a significant decreased 24-h urine creatinine and creatinine/kg body weight ratio during follow-up was detected, thus suggesting, despite the weight gain, a potential muscle mass loss as 24-h urine creatinine is currently viewed as a biomarker.

The comparison of the 24-h urine ionogram between the three visits showed urine output of more than 2 L per day, with no significant change over time (Table 3). As shown, urine also provides information about dietary sodium intake and protein load. Urine sodium expressed as sodium chloride intake was 8.3 g/day at baseline with 46% of patients within the recommended targets versus 52% of patients at visit 3, thus showing no significant changes despite dietary advice (*p* = 0.22). Daily protein intake was high with a median value of 1.29 g/kg/day at baseline and decreased significantly thereafter (40.3% and 45.9% of patients below 1 g/kg/day intake at baseline and last visit, respectively, *p* = 0.001). Restricted protein diet improvement was associated with decreased osmoles excretion and ammonium excretion (Table 3), whereas daily urine phosphate was similar throughout follow up. Of notice, daily calcium excretion also decreased during follow up from 1.47 to 1.33 mmol/day (*p* = 0.03).

## 4. Discussion

To our knowledge, this is the first report evaluating in a non-end stage CKD cohort the reliability and interest of the 24-urine ionogram data in order to monitor dietary targets during a two year follow-up.

Several studies suggest the interest of spot urine collection because of the difficulty of having a 24-h urine collection [19,22,23] with no or few available data evaluating the validity of the collection [21]. If urine albumin or protein to creatinine ratio are reported to be a reliable substitute for 24-h proteinuria and albuminuria in CKD [24,25,26,27], accuracy is poor for the evaluation of food intake [28,29] and 24-h urine sodium rather than a spot urine sample would be considered as the gold-standard [30,31].

Validation of true 24-h urine collection is indeed the main concern as it may under or overestimate osmoles and water intake (if we consider no relevant extra renal wasting). We raised the issue of whether 24-h urine creatinine expressed in µmol/kg body weight, could be used in order to validate 24-h urine collection, despite well-known pitfalls such as muscle mass loss during CKD progression. Indeed, daily urine creatinine excretion was previously shown to parallel with GFR decrease (0.28 ± 0.02 mmol/24 h for a GFR decrease of 1.53 ± 0.12 mL/min/1.73 m^2^), suggesting variation over time due to muscle mass loss [20,21]. Moreover, our data showed weight gain during follow-up (+7% in two years), also suggesting either increased fat mass or a fluid overload [32], a reported CKD progression risk factor [33], which will further decrease the 24-h urine creatinine/body weight ratio during follow-up. Nevertheless, despite the scarcity around median values reported for this parameter [21] and also shown also in our study (Table 2), the 24-h urine creatinine/body weight ratio seemed to be a rather robust marker for 24-h urine collection validation with 58% of valid collection at the baseline visit as compared to 61% using fractional creatinine clearance.

We took advantage of the fractional creatinine clearance, i.e., the mean creatinine clearance performed on six spot urine samples during patient outpatient hospitalization, to postulate that the 24-h urine collection was valid if the difference with the 24-h creatinine clearance was less than 30%. Based on this threshold, our data show that among the 404 patients included initially, 5% had no valid 24-h urine collection at any visit, 65% had a valid 24-h urine collection on the first evaluation. This percentage rose to a striking 78% of reliable urine collection after the third visit. By decreasing the threshold down to 15%, we found 51% of valid urine collections at the first visit and 67% at third visit, thus suggesting that osmoles and water intake may be reliably estimated. Interestingly, the assessment of valid urine collection was about the same order of magnitude when the 24-h creatinine clearance was compared to the eGFR formula instead of the fractional creatinine clearance, (53% and 65% at the baseline visits, respectively, for a 30% threshold), thus suggesting that reliable 24-h urine collection is a goal achievable in more than half of patients in daily practice.

Among the 168 patients with three valid 24-h urine collections, our data provide interesting information about diet over time allowing the monitoring of water intake estimates with diuresis above 2 L/day. Indeed, increased diuresis may be a target in some renal diseases such as Polycystic Kidney Disease (PKD) [34,35]. As shown Table 3, other dietary targets such as salt intake were achieved in 51.6% of patients at the first visit with a median value of 8.3 g/day, whereas protein intake estimates were high (>1 g/kg body weight/day) at the first visit in 77.4% of patients and significantly decreased from a median value of 1.29 to 1.18 g/kg/day at visit 3, thus accounting for a significant decrease of daily osmoles intake and urine ammonium excretion. Indeed, lowering protein diet is expected to decrease the net acid load [36] but also phosphate excretion conversely to our data. This finding may suggest other dietary sources of phosphate such as appetizing factors, taste enhances, and consumption of large amounts of high phosphate sodas.

Of notice, among our population with two years follow-up, mGFR significantly decreased from 43.7 to 38.5 mL/min/1.73 m^2^ (*p* < 0.0001). During this period, the prevalence of secondary hyperparathyroidism increased significantly from 26.2 to 31.5% with no change in ionized calcium, serum phosphorus, and calcitriol, thus suggesting that calcium intestinal absorption may have decreased. As a matter of fact, daily urine calcium excretion decrease was significant though moderate, thus suggesting that body calcium needs were at least partly fulfilled at the expense of PTH increase in some patients, thus encouraging further calcium intake increase or calcium prescription in order to prevent secondary hyperparathyroidism. According to this view, monitoring calciuria and defining a specific target to prevent hyperparathyroidism in CKD patients could be an interesting approach and should deserve further interventional studies.

Limitations of our study: The aim of the present study was to focus on 24-h urine ionogram reliability in a CKD population and to illustrate the interest of urine data to estimate diet intake and patient’s compliance to dietary targets. The effectiveness of dietary targets, minerals, and vitamins supplements in our population is, thus beyond the focus of the present study and should deserve to be specifically addressed. Once 24-h urine collection is considered valid, interpretation of the data from the urine ionogram provides an estimate of ongoing diet and does not preclude significant variations depending on the day of the week or seasons... Moreover, diet estimation based on 24-h urine collection implies that the patient is in a steady-state, and thus the interpretation of urine data should be cautious, noteworthy in patients with bowel diseases.

To conclude, our study shows that 24-h urine ionogram appears as an interesting reliable tool in 65–78% of CKD patients to evaluate food intake, providing interesting information in addition to the diet survey to achieve KDIGO recommendation targets of salt and protein intake [7]. Once urine collection is validated after comparison of the 24-h creatinine clearance to either eGFR or creatinine excretion normograms [21], it may also provide an interesting tool for patient education and diet compliance, daily calcium needs but also nutritional status, especially muscle mass during follow-up. 

## Figures and Tables

**Table 1:** Comparison of the patient’s biological data according to 24-h urine collection compliance.

Biological Data	Patients with 3 Valid 24-h Urine Collections	Patients with No Valid 24-h Urine Collections	*p*-Value
***n* =**	168	22	
**Blood**			
mGFR (mL/min/1.73 m^2^)	43.7(31.3–56.4)	40.3 (19.5–53.8)	0.2
Creatinine Clearance (mL/min)	56.9(45.2–70)	62 (37.7–82.2)	0.84
Serum creatinine (µmol/L)	134 (107.3–172)	126.6 (95.5–190.8)	0.98
Natremia (mmol/L)	141 (139–142)	141 (140–142)	0.37
Serum potassium (mmol/L)	4.2 (4.2–4.5)	4.1 (4–4.3)	0.17
Hemoglobin (g/dL)	12.8 (11.8–14)	12.4 (11.6–13.9)	0.59
Ionized calcium (mmol/L)	1.19 (1.16–1.21)	1.19 (1.15–1.22)	0.84
PTH (pg/mL)	43 (30–71.5)	45 (34–96)	0.24
**Urine**			
Diuresis (mL/Day)	2175 (1716–2700)	1657 (1350–2207)	0.003
Proteinuria (mg/mmol creatinine)	19.1 (3–88.6)	17.1 (5–73)	0.42
ACR (mg/mmol creatinine)	5.1 (1.7–38.4)	10.3 (2.9–40.1)	0.37
Creatinine (mmol/day)	13.1 (9.5–17.3)	8.2 (5.7–13.3)	0.008
Creatinine (mmol/kg/day)	0.17 (0.13–0.23)	0.10 (0.07–0.18)	0.004

**Table 2:** Comparison of the clinical and biological parameters between the three visits in patients with all validated 24-h urine collections (*n* = 168).

Parameters	Visit 1	Visit 2	Visit 3	*p*-Value
Blood pressure > 130/80 mmHg (%)	54.5	57.6	62.6	0.12
Weight (kg)	70 (63–85)	74 (64–84)	75 (65–85)	0.07
**Blood**				
mGFR mL/min/1.73 m^2^)	43.7 (31.3–56.4)	41.2 (29.1–55.5)	38.5 (25–52.2)	<0.001
Creatinine Clearance (mL/min)	56.9 (45.2–70)	55.9 (40.6–72.6)	51.6 (36.2–68.9)	<0.001
Serum creatinine (µmol/L)	134 (107.3–172)	137.5 (106.5–178.5)	136.8 (106.4–190.3)	<0.001
Haemoglobin (g/dL)	12.8	12.7	12.6	0.10
Serum potassium (mmol/L)	4.2 (4.2–4.5)	4.2 (3.9–4.6)	4.2 (4–4.6)	0.24
Bicarbonate (mmol/L)	26.3 (24.3–28)	26.5 (24.9–28.5)	26.3 [24.5–28.1)	0.37
Phosphorus(mmol/L)	1.03	1.03	1.06	0.18
Ionized calcium (mmol/L)	1.19 (1.16–1.21)	1.19 (1.16–1.22)	1.19 (1.16–1.22)	0.06
PTH (pg/mL)	43 (30–71.5)	48 (2.3–80.8)	50 (36.5–85.3)	<0.001
Secondary hyperparathyroidim (%)	26.2	29.3	31.5	<0.001
25-OH-VitD (ng/mL)	26.8 (17–35.7)	27.3 (19–36)	24.8 (18.3–32.9)	0.91
Calcitriol (pg/mL)	34 (22.1–43.9)	33 (22.6–44.4)	33.2 (24–43.6)	0.98
**Urine**				
Proteinuria (mg/mmol creatinine)	19.1 (3–88.6)	22 (6.8–77.8)	19.2 (3–88.6)	0.84
ACR (mg/mmol creatinine)	3.4 (1.3–27.4)	4.6 (1.4–28.2)	5.1 (1.7–38.4)	0.005
Creatinine (mmol/kg/day)	0.17 (0.13–0.23)	0.15 (0.12–0.18)	0.14 (0.12–0.17)	<0.0001

**Table 3:** Comparison of dietary data obtained from 24-h urine in patients with validated 24-h urine collections (*n* = 168).

Dietary Data	Visit 1	Visit 2	Visit 3	*p*-Value
Urine volume (mL/day)	2175 (1716–2700)	2203 (1639–2743)	2159 (1778–2794)	0.80
Estimated sodium chloride intake (g/day)	8.3 (6–10.8)	7.8 (5.4–10.7)	7.7 (5.8–9.6)	0.25
Estimated protein intake (g/kg/day)	1.29 (1.05–1.47)	1.23 (0.96–1.42)	1.18 (0.95–1.41)	0.02
Estimated Osmole intake (osm/day)	779.9 (655–935)	745.2 (592–889)	740.4 (616–857)	0.006
Phosphate excretion (mmol/day)	20.1 (14.9–27.8)	20.5 (13.3–30.4)	20.1 (14.4–27.6)	0.18
Calcium excretion (mmol/day)	1.47 (0.81–2.78)	1.52 (0.66–2.80)	1.33 (0.61–2.53)	0.03
Ammonium excretion (mmol/day)	20.2 (13.1–31.7)	18.3 (11.1–27.3)	17.6 (11.3–26.5)	0.004
